# A Comprehensive Review on Bio-Preservation of Bread: An Approach to Adopt Wholesome Strategies

**DOI:** 10.3390/foods11030319

**Published:** 2022-01-24

**Authors:** Mizanur Rahman, Raihanul Islam, Shariful Hasan, Wahidu Zzaman, Md Rahmatuzzaman Rana, Shafi Ahmed, Mukta Roy, Asm Sayem, Abdul Matin, António Raposo, Renata Puppin Zandonadi, Raquel Braz Assunção Botelho, Atiqur Rahman Sunny

**Affiliations:** 1 Department of Food Engineering and Tea Technology, Shahjalal University of Science and Technology, Sylhet 3100, Bangladesh; raihanul08@student.sust.edu (R.I.); sharifulh.nahid@gmail.com (S.H.); wahid-ttc@sust.edu (W.Z.); rzaman-fet@sust.edu (M.R.R.); muktaroy-fet@sust.edu (M.R.); asm.sayem-fet@sust.edu (A.S.); 2 Department of Agro Product Processing Technology, Jashore University of Science and Technology, Jashore 7400, Bangladesh or shafi@just.edu.bd; 3 Department of Food Processing and Engineering, Chattogram Veterinary and Animal Sciences University, Chittagong 4203, Bangladesh; abmatin@cvasu.ac.bd; 4 CBIOS (Research Center for Biosciences and Health Technologies), Universidade Lusófona de Humanidades e Tecnologias, Campo Grande 376, 1749-024 Lisboa, Portugal; 5 Department of Nutrition, Campus Darcy Ribeiro, University of Brasilia, Asa Norte, Distrito Federal, Brasília 70910-900, Brazil; renatapz@unb.br (R.P.Z.); raquelbotelho@unb.br (R.B.A.B.); 6 Department of Genetic Engineering and Biotechnology, Shahjalal University of Science and Technology, Sylhet 3100, Bangladesh; 7 Suchana Project, WorldFish, Bangladesh Office, Gulshan, Dhaka 1213, Bangladesh

**Keywords:** bakery products, bio-preservatives, shelf-life, mold spoilage, antifungal activity, microbial fermentation, active packaging

## Abstract

Bread is a food that is commonly recognized as a very convenient type of food, but it is also easily prone to microbial attack. As a result of bread spoilage, a significant economic loss occurs to both consumers and producers. For years, the bakery industry has sought to identify treatments that make bread safe and with an extended shelf-life to address this economic and safety concern, including replacing harmful chemical preservatives. New frontiers, on the other hand, have recently been explored. Alternative methods of bread preservation, such as microbial fermentation, utilization of plant and animal derivatives, nanofibers, and other innovative technologies, have yielded promising results. This review summarizes numerous research findings regarding the bio-preservation of bread and suggests potential applications of these techniques. Among these techniques, microbial fermentation using lactic acid bacteria strains and yeast has drawn significant interest nowadays because of their outstanding antifungal activity and shelf-life extending capacity. For example, bread slices with *Lactobacillus plantarum* LB1 and *Lactobacillus rossiae* LB5 inhibited fungal development for up to 21 days with the lowest contamination score. Moreover, various essential oils and plant extracts, such as lemongrass oil and garlic extracts, demonstrated promising results in reducing fungal growth on bread and other bakery products. In addition, different emerging bio-preservation strategies such as the utilization of whey, nanofibers, active packaging, and modified atmospheric packaging have gained considerable interest in recent days.

## 1. Introduction

For thousands of years, bread is still one of the dominant food sources of the human diet, with the manufacturing of yeast-based and sourdough bread being one of the earliest biotechnological mechanisms [1]. Amidst its medium growth rate (122,000 t in 2007 to 129 t in 2016), it earned approximately $358 billion in global revenue in 2016 [2]. It is also a magnificent energy source, including protein, iron, calcium, and various vitamins [3,4,5,6]. Commercially available bread and biscuits contain nearly 7.5 and 7.8% protein [7]. Bakery products are ideal for fiber addition, as fiber intake has declined in the European diet partially due to cereal adjustments.

Due to the easily spoiled nature of this food, its quality and palatability degrade during preservation, resulting in changes in physiological, biochemical, sensorial, and microbial properties [8]. Mold and fungal deterioration are the primary causes of significant financial detriments in packed bread items. They may also be considered as mycotoxins sources [9], posing issues to the people′s health [10,11,12,13] and putting a significant financial strain on bakeries. Penicillium (*Penicillium chrysogenum*, *Penicillium roqueforti*, and *Penicillium brevicompactum*), Aspergillus (previously Eurotium), Wallemia, and other familiar molds such as Rhizopus, *Chrysonilia sitophila*, and Mucor are the main genera involved in the spoiling of bakery items [14,15,16]. Yeasts, specifically *Saccharomycopsis fibuligera* and *Hyphopichia burtonii*, can also contribute to the “chalk mold” problem [7]. Due to these microbial attacks, a high proportion of food waste is being produced around the globe. For example, in Germany in 2015, 34.7% of total bread was lost [17]. Article [18] estimated losses of 10% in Brazil and presumably other nations with a tropical environment. Additionally, mycotoxin infection in food items is claimed to be a universal issue [19,20,21,22] and it occurs in nearly 25% of all grain yields globally [23]. A study conducted in Poland identified nine categories of causes of losses based on a quantitative study within the four considered sectors of the bakery enterprise: (i) raw materials magazine—mechanical damage, magazine pests, spoiling, molding, and impurities; (ii) production section—hygiene and sanitary requirements, technical breakdowns; (iii) final product magazine—damaged packaging, hygiene and sanitary requirements/food safety hazards, technical breakdowns; and (iv) final product transport—errors in placed orders, damaged unit packaging, technical breakdowns, incomplete collective packaging [24]. In all of them, mold and fungal deterioration may cause losses.

Today’s food industry faces a tremendous problem in producing goods that are not only productive but also wholesome for customers, as well as much more long-lasting. The use of organic preservatives has the feasibility of meeting both needs [25,26]. Natural antimicrobial preservatives have been the subject of extensive research due to the growing evidence of the harmfulness of chemical preservatives and their impacts on consumer health [27,28,29]. The restoration of chemical preservatives—such as propionates and sorbates—in bread and other bakery goods is of considerable interest [25].

Chemical preservatives, such as calcium propionate, are commonly applied to expand the microbial lifespan of bread [30,31]. However, prolonged exposure to chemical preservatives may pose a health risk. Thus, using bio-preservation techniques on bread can aid in solving this issue and preventing economic loss caused by fungi, mold, or yeast [32]. Bio-preservatives are organic resources that can be utilized to lower or eliminate microbial populations while improving food quality. It is a revolutionary concept for processing and preserving perishable fresh goods and is generally recognized as safe (GRAS) for food usage. Numerous distinct studies have been conducted on bio-preservation. Thus, the objective of this review was to integrate all studies conducted on the spoilage problem of bread, as well as the preservation techniques, most notably bio-preservation, used to extend its shelf life.

## 2. Materials and Methods

A search strategy was conducted on key literature databases such as SCOPUS, PubMed, ResearchGate, and Google Scholar. The keywords mentioned in Table 1 were used to find articles, starting with the primary keywords, then joined with the secondary keywords using the set operator AND. Article references were also reviewed to evaluate other relevant publications.

The studies related to the spoilage and bio-preservation of bread were included in this review. Additionally, some supporting facts about the varieties and economic relevance of bread was added. It summarized only previously conducted, scientifically validated findings. Articles published in regional languages have been excluded entirely. Papers that lacked sufficient data on the subject or failed to adequately characterize the impact of bio-preservatives on bread were omitted entirely. This topic does not include articles on chemical preservation of bread, nutritional analysis, bread formulation and innovation, or functional aspects of bread. No papers with inconclusive outcomes were considered for this article. Finally, to study this clean-level alternative, only works focusing on recent advances in this field were cited, with the exception of a few fundamental principles.

## 3. Bread and Its Types

The world’s expanding, aging, more urbanized, and nomadic population calls for a more accessible, nutritious, and safe food supply. Consuming wholemeal or multigrain bread may provide the most outstanding contribution to the necessary nutritional intake [33]. It is remarkable that white bread now surpasses pasta, pseudocereals, rice, and tubers in terms of consumption.

**Figure 1:**
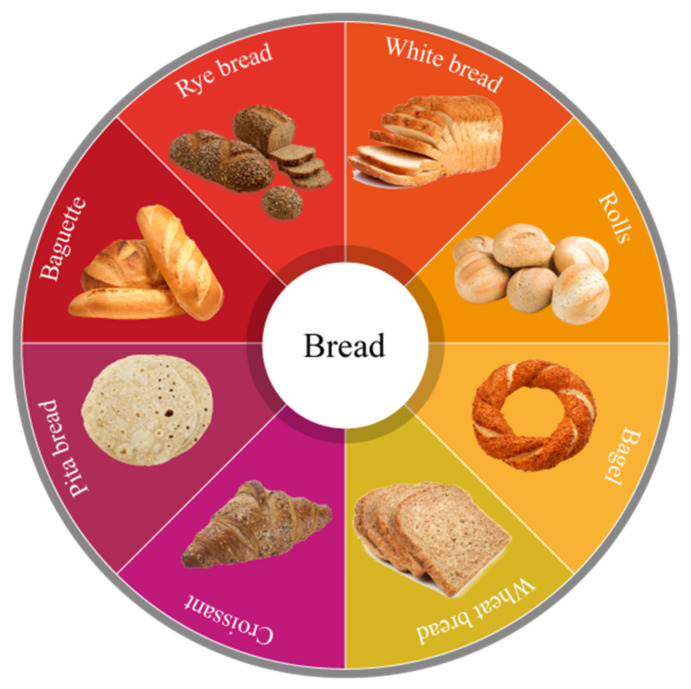
Different types of bread around the world.

There are several varieties of bread (Figure 1), with national and regional variants on fundamental types and an abundance of specialty varieties, such as malt loaves, soda loaves, milk loaves, fruit loaves, and cream loaves [34]. Additionally, there is high protein bread, added malt grain bread, high fiber, multigrain bread, wheat germ bread, soft grain bread, ethnic multigrain bread, slimming and healthy high fiber bread, bread for special dietary needs, and cereal bread other than wheat crispbread. The bakery industry does not use the same terminology to describe bread and its quality characteristics, leading to misunderstandings regarding bread quality among consumers.

The most prominent example references French bread, which in the United Kingdom, is often referred to as baguette-style bread. In contrast, in the United States, the word may apply to pan bread with lean (i.e., low-fat) formulas, such as those adopted to make French toast. The term “French” bread is not as well understood in France as in the United Kingdom. Correspondingly, the conception of “toast bread” has a distinct connotation in France, the United States of America, the United Kingdom, and elsewhere [35]. The description of a specific bread type will always explain its substantial look, most often with its outer structure. Consequently, the length and diameter of baguettes are often used to classify them, whereas the pan form of other bread is used to classify Middle Eastern and conventional Indian bread. Even surface markings may require definition, as the quantity and pattern of cuts on the dough surface may become an essential aspect of the traditional appearance of the product.

## 4. Economic Importance of Bakery Product

Bread is consumed in a diversity of shapes and forms all over the world, with an average annual consumption of 70 kg per capita; however, Europe consumes less bread, with an average annual consumption of 59 kg [36,37,38]. Bread products have evolved into various shapes and flavors, each with its own unique set of features. It is more favorable in an urbanized environment because it requires no additional preparation and is immediately consumable. Bread consumption has already increased in areas where it was previously considered a luxury item. For example, between 1980 and 2008, per capita wheat consumption increased by 44% in Africa due to its convenience and ease of preparation [39]. Annually, almost 9 billion kg of bread items are manufactured [40], with an expected annual per capita intake of 41 to 303 kg [41]. Moreover, Asians are increasing their bread consumption. The average volume of bread and bakery products consumed per person is predicted to reach 120.5 kg in 2021 and an annual growth of 4.87% is estimated from 2021 to 2026. Globally, the majority of income is earned in China (US $275,427 million in 2021) [42]. It is worth noting that India produced 257 thousand metric tons of baked goods in fiscal 2020, compared to 275 thousand metric tons in fiscal 2018 [43] in Europe; the consumption of bread and bakery products climbed from 29,641 million kg in 2010 to 30,328 million kg in 2017 and is predicted to reach 30,352 million kg in 2021 [44]. In Chile, people consume about 86 kg per capita/year of bread, the highest per capita among Latin American countries. Argentina presents the second-largest consumption at 50 kg per capita/year [45]. In Brazil, bread consumption is about 34 kg per capita/year.

## 5. Spoilage Concerns of Bread

Bread quality deteriorates after baking, resulting in significant monetary losses for the bakery sector and the customer [46]. Bread spoilage is a complicated process that undergoes chemical (changes in nutraceutical value, rancidity), physical (moisture redistribution, staling), and microbiological (yeast, bacterial spoilage, and mold) alterations and contributes to the “staling process” of bread [11,47]. Staling shortens the shelf life of bread, which is determined as the period that food remains “acceptable” for consumption under certain storage conditions. Acceptable means retaining the desired sensory, chemical, physical, and biological attributes in addition to being safe [48]. The other significant reason for the reduction of the shelf life of bakery goods through post-baking storage is microbial spoilage, which ultimately results in detectable mold growth and the generation of mycotoxins that are not identifiable. High moisture levels (a_w_ = 0.94–0.99) stimulate the development of approximately all bacteria, yeasts, and molds [49,50,51,52]. We will review some of the most familiar concerns about bakery spoilage in the following sections.

### 5.1. Physical Spoilage

Moisture absorption and loss can cause physical changes in low and intermediate moisture products, as well as chemical and microbiological deterioration. As well, the staling of bakery items is the most severe issue with physical deterioration [51]. The total staling mechanism involves two distinct phenomena: the intrinsic firming of the cell wall substance related to starch re-crystallization at the time of storage and the firming effect induced by moisture migration from crumb to crust [17,53]. It has an eloquent economic impact on the baking business. Furthermore, bread loses nutritional value and chemical stability after baking, usually linked with rancidity, particularly in bread processed with whole wheat flour or possessing a high-fat content [54]. By breaking down unsaturated fatty acids via malodorous aldehydes, autolytic free radicals, short-chain fatty acids, and ketones are combined, leading to rancidity [55].

### 5.2. Chemical Spoilage

Rancidity is the most common type of chemical spoilage that occurs after baking high-fat bread products [54]. It is known as lipid degradation, and it results in off-odors and flavors. Rancidity lessens the bread′s shelf life and makes it highly unhealthy for consumers. There are two different types of rancidity in general: oxidative and hydrolytic rancidity [51].

Oxidative rancidity induces the degradation of unsaturated fatty acids by oxygen using an autolytic free-radical process. As a result, foul-smelling aldehydes, ketones, and short-chain fatty acids are produced. These free radicals and peroxides developed by lipid oxidation may also negatively impact food composition by bleaching pigments (for example, lycopene in tomato paste in pizza), deteriorating protein, and spoiling specific vitamins (for example, vitamins A and E). In contrast to oxidative rancidity, hydrolytic rancidity develops without oxygen triglyceride hydrolysis and the consequent discharge of malodorous fatty acids and glycerol. Moisture and endogenous enzymes such as lipases and lipoxygenases trigger rancidity problems.

### 5.3. Microbiological Spoilage

Bread ingredients promote the growth and proliferation of microbes during various phases of bread preparation, processing, packing, and storage. Study [56] found that molds, yeasts, and bacteria were the primary sources of microbiological spoilage in bread, and that by using cluster analysis, the enzymic degradation induced by lipoxygenase may be separated from each other and preserve bread analogs after 48 h, prior to apparent indications of decomposition. They can survive in the baking environment and multiply in various conditions, including those where other bacteria are not competitive [57].

Water activity (a_w_) has the most significant impact on the microbial spoilage of bakery items. Microbiological spoilage is not an issue with low moisture bread items (a_w_ 0.6). The primary spoilage pathogens in moderate moisture (aw 0.6–0.85) are osmophilic yeasts and molds. Mainly all yeasts, molds, and bacteria can thrive in high-moisture conditions (a_w_ 0.94–0.99) [58,59].

#### 5.3.1. Bacterial Spoilage

Spore-forming bacteria are another source of concern for bread quality and safety. This problem is specific to bakery goods with a high moisture content as most bacteria need high a_w_ to survive [51]. The key pollutants are ingredients and/or bakery equipment, as spore-forming bacteria are almost certainly produced on the extraneous surface sections of grains and in the surrounding air of the bakery [60].

*Bacillus subtilis* is the main cause of bacterial spoilage. Its spores form endospores, which possibly sustain baking and sprout and develop inside the bread within 36–48 h, forming the distinctive soft, fibrous, brown mass with a ripe pineapple or melon odor leading to the exoneration of volatile components, for example, diacetyl, acetoin, acetaldehyde, and isovaleric-aldehyde [53]. *Bacillus subtilis* spores are heat-resistant; after 20 min at 65 °C, 55% remain active in amylase [7]. Other Bacillus species have been identified that cause bacterial spoilage of bread, including *Bacillus amyloliquefaciens*, *Bacillus pumilus*, *Bacillus licheniformis*, *Bacillus megaterium*, and *Bacillus cereus* [60] (Table 2). According to [61,62], *B. amyloliquefaciens* could be the primary species responsible for rope spoilage. “Ropey” bread can be distinguished by brown to black discoloration, the release of a rotten fruit odor, and the formation of sticky breadcrumbs as a result of protein and starch degradation during bacterial growth [63,64].

#### 5.3.2. Fungal Spoilage

**Figure 2:**
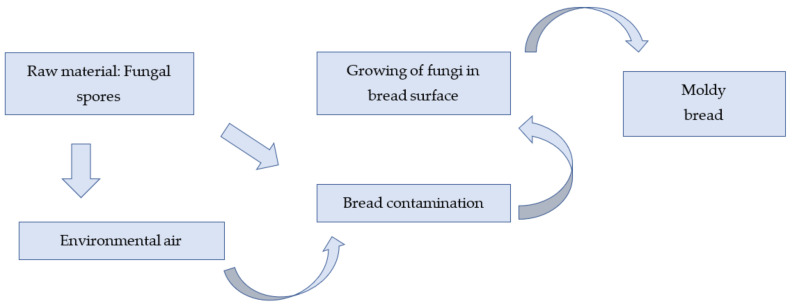
Fungal spoilage procedure. Adapted from ref. [73].

Wheat, rice, and maize are the three most significant cereal crops grown globally [63]. In the Western world, wheat is the most considerable cereal for making bread, accompanied by rye. Barley, oats, and the gluten-free crops quinoa, millet, sorghum, buckwheat, and amaranth are all important cereals [46,63]. The contamination of cereal grains and their products occurs after harvest, during storage time, and pre- and post-processing. An estimated 40 species of fungi have been discovered in baked goods, most of which are considered contaminants [64]. Among these, Penicillium, Aspergillus, Rhizopus, and Wallemia are the most prevalent fungi species liable for the fungal deterioration of bread [8,14,15]. An illustration of fungal spoilage is shown in Figure 2. Some of the fungal spoilage types are discussed below:

1. Mold Spoilage: Mold in bread is a serious concern as it creates mycotoxin, which is harmful to public health [65,66,67]. Mold growth and mycotoxin production in crops and foods are challenges in emerging countries in Africa and Asia, leading to increased humidity and temperature [68,69]. It also shortens the shelf-life of bakery items containing moderate and high moisture content. Most molds can generally grow at a_w_ values greater than 0.80, except for a few xerophilic molds, which may thrive at a_w_ values as low as 0.65. While baking, molds and spores may be rendered inactive. However, since the environment within a bakery are not hygienic and might be a source of contamination, the mold contamination of bread can occur during chilling, slicing, wrapping, and storage [53]. Moisture precipitates on the inner area of the package when hot bread is wrapped, promoting mold growth. The most frequent molds involved in bread spoilage are Rhizopus nigricans, which has fluffy white mycelium and black spots of sporangia, and *Penicillium expansum* or *P. stolonifer*, and *Aspergillus niger*, which has greenish to black conidial heads and are referred to as “bread mold” [53,70,71] (Table 2). Aspergillus, Mucorales, Cladosporium, Penicillium, and Neurospora species have all been found in wheat bread, with *Penicillium* spp. being the most frequently occurring variety [11].
2. Yeast Spoilage: Yeast spoilage is the type of microbial spoilage with the lowest prevalence. It occurs most frequently during cooling, and more significantly, during the slicing stage in the case of industrial bread. Off-odors in bread may indicate yeast spoilage. Yeast spoilage can occur in two different forms of yeast [72]. (a) An alcoholic or esteric off-odor is generally identified when fermentative yeasts cause spoilage. Most seen is *Saccharomices cerevisiae*, which is commonly employed as baker′s yeast. (b) The other type of contamination is due to filamentous yeasts. The familiar circumstance described as “chalky bread” develops in this situation, showing white dots in the crumb. Mold growth is sometimes confused with this type of spoilage. However, yeasts create single cells and reproduce through budding; thus, they can be differentiated. *P. anomala*, *Scopsis fibuligera,* and *H. burtonii* promote the prime spoilage of bread goods by growing in white, low, spreading colonies that symbolize a scattering of chalk dust on the product surface. Visible yeast generation is typically linked with goods with a high a_w_ and short shelf life.

## 6. Bio-Preservation to Control the Spoilage of Bread

The bakery sector uses product reformulation, which is the most popular, practical, and cost-effective solution to prevent post-baking contamination. This is done by lowering the product’s a_w_ and pH, which are related to the microorganisms’ shelf life. They also use chemical preservatives directly in the product or on its surface to prevent bacterial and microbial spoilage [51]. According to [74], chemical preservatives inhibit microbial metabolism by denaturing cell protein or causing physical damage to the cell membrane. Propionic acid, as well as its salt, are the most widely used chemical preservatives in bread [7]. It helps prevent mold deterioration and bread ropiness that occurs due to *B. subtilis*.

However, they are continually investigated due to the possibility of developing chronic non-communicable diseases [75]. As a result, bio-preservatives have emerged as a favored solution to these shortcomings, with the intention to generate “clean label” foods [15,76,77]. Bio-preservatives can also be adopted as natural antifungal substances to prevent fungal degradation and prolong shelf life, reducing public health risks [2,12]. Bio-preservatives such as lactic acid bacteria, essential oils, or natural nanoparticles are becoming more popular because of consumer apprehensions about applying chemicals in food. According to [78,79,80,81,82,83], a good bio-preservative should have the following characteristics: it should have an expansive antibacterial spectral range, be non-toxic to humans, be suitable for lower doses, have some slight impact on product pH, not impair product odor, color, or flavor at the proposed level of its use, be accessible in a dry state, have a higher water solubility, be non-corrosive, be unreactive, and have no detrimental effects on fermentation or bread character. We will review a few of the bio-preservatives in the following sections.

### 6.1. Microbial Fermentation

Traditionally, organic and flavorful bread with a long shelf life was achieved naturally through an expanded fermenting operation: sourdough [11,84,85,86,87,88,89]. The word “sourdough” describes a particular bread recipe in which flour, water, LAB, and yeast organisms are fermented together [89,90,91,92,93]. Because of their remarkable antifungal activity, lactic acid bacteria (LAB) and antagonistic yeasts have received particular attention among natural agents [93,94,95,96,97] and are herein discussed below as well as presented in a tabular form (Table 3).

Lactic acid bacteria: LAB metabolic products enhance bread’s organoleptic and technological aspects, as well as its textural characteristics [81,82,112,113,114,115,116], along with its shelf life, nutritional value [83,117,118,119,120,121], and beneficial aspect (anticarcinogenic and cholesterol reduction abilities), during the fermentation of the dough [84,85,86,122,123,124,125,126]. They can also be adopted to replace chemical preservatives, ensuring a clean label and increased consumer acceptance [87,126,127,128,129].

Lactic acid bacteria (LAB) have been utilized in fermented foods for over about 4000 years [88]. It is naturally found in foods or introduced as pure cultures [89]. It is also GRAS-certified (generally recognized as safe) and has an extended application history in various cereal fermentations, particularly in the baking industry. LAB’s adaptability is remarkable, not just in terms of catabolic and anabolic pathways but also in changing environmental conditions [90,130,131,132,133,134].

It has also been employed as a starter culture in the food business for centuries, which may produce several bioactive compounds, along with fatty acids, bacteriocins, organic acids, hydrogen peroxide, indole lactic acid, and phenyl lactic acid [91]. They also have an anti-aflatoxigenic effect [92,135,136,137,138]. Particular lab strains that have gripping bio-preservation action on bread when adopted as starter cultures include *Lactobacillus amylovorous* DSM 19,280, *Lactobacillus fermentum* Te007, *Lactobacillus acidophilus* ATCC 20079, *Lactobacillus paralimentarius* PB127, *Lactobacillus brevis* R2D, *Lactobacillus rossiae* LD108, *Lactobacillus hammesii*, *Lactobacillus paracasi* D5, *Pediococcus pentosaceus* KTU 05-8 and KTU 05-10, *Lactobacillus pentosus* G004, *Lactobacillus plantarum*, *Lactobacillus reuteri* R29, *Lactobacillus rhamnosus*, *Lactococcus* BSN, *Pediococcus acidilactici* KTU05-7, as well as *Leuconostoc citreum* C5 and HO12 [93,139,140,141,142]. Additionally, adding 15–20% sourdough significantly (*p* = 0.0001) increased bread volume and crumb porosity, based on the LAB strain, and reduced acrylamide formation by an average of 23% (for LUHS51 and LUHS206) and 54% (for LUHS71 and LUHS225), respectively, in comparison with regular bread [94]. Also, the most dominating species of the conventional sourdough microbiota, *Lactobacillus sanfranciscensis*, has been found to have a favorable impact on several important quality features of sourdough, notably dough rheological qualities, bread texture and aroma, and shelf-life conservation [95,96,143,144,145].

According to [97], LAB mix culture-activated bread samples could tolerate fungal deterioration until the fourth day. It was also discovered that the primary products of LAB fermentation, such as lactic and acetic acid, inhibited further fungal growth in *Mucor* sp. and *Rhizopus* sp. by up to 40% and 20%, respectively, when compared to a control bread sample. Also, the development of *A. niger* was observed in a study by [98,146]. They found that its growth in pan bread containing LAB isolate was slower than the control bread without the isolate after 7 days of baking. Moreover, the antifungal efficacy of *Lb. amylovorus* DSM19280 as a sourdough starter culture was evaluated by [99]. The result showed that, when it was applied, the bread’s shelf life was increased by 4 days, relative to the control samples, which had mold detectable after only 2 days. Moreover, [100,147] used *P. acidilactici* KTU05-7, *P. pentosaceus* KTU05-8, and KTU05-10 strains on sourdough bread in another experiment. Their findings showed that adding sourdough made with these strains in bread decreased fungal deterioration more than control samples and suppressed fungal growth over an 8-day storage period, whereas control bread had visible fungi colonies. Furthermore, to investigate the antifungal activity of *Lactobacillus rossiae* LB5 and *Lb. plantarum* LB1, bread slices with *Lactobacillus rossiae* LB5 and *Lb. plantarum* LB1 were mixed with *Penicillium roqueforti* DPPMAF1 by [101,148]. Mycelial development was seen in the wheat germ bread sample after 21 days of inoculation with only a 10% contamination score. In comparison to control samples injected with *Cladosporium* spp., *Aspergillus clavatus*, *Penicillium roquefortii*, or *Mortierella* spp., [102] studied the implications of using two active propionate providers, *Lactobacillus diolivorans* and *Lactobacillus buchneri*, in bread restoration, and discovered that mold development was inhibited for more than 12 days.

- Yeast: Numerous authors have experimented with using incompatible yeasts as biocontrol agents. They can be used as bio-preservatives as they retain some of the essential features that enhance their acceptability. They compete for nutrients with fungal pathogens and their higher rate of nutrient utilization significantly contributes to a bio-preservative nature. Yeast produces killer toxins, also called mycotoxins, which showed bioprotective attributes against food spoiling microorganisms and pathogens [149]. Some yeast genera produce extra- and intracellular compounds which possess antibacterial properties. Production of ethanol of high concentration and organic acids which results in the change of pH of the medium also responsible for the effectiveness of yeasts as bio-preservatives [150]. Many of them can sustain residence on dry surfaces due to their low requirements for water and nutrients [103]. With *Lb. plantarum* 1A7 as a starter, the yeast *Wickerhamomyces anomalus* LCF1695 (previously recognized as *Pichia anomala*) was occupied for sourdough fermentation. It was found that when *P. roqueforti* DPPMAF1 was artificially inoculated (102 conidia ml1) with this combination and stored at room temperature, the microbiological shelf life was elongated to at least 14 days [104]. As well as *Penicillium paneum* KACC 44834, outgrowth on white pan bread leavened with *Penicillium anomala* SKM-T was significantly reduced compared to standard baker′s yeast, and improved the shelf life [105]. In contrast, bread composed of propionic acid from cultured yeast extract contained less ethanol and had a better shelf life against mold growth than bread formulated with non-fermented yeast extract [106].

### 6.2. Plant Extracts as Bio-Preservative

Plant extracts have been extensively studied as bio-preservatives, as plants contain many essential antifungal compounds, for example, phenolic compounds, glucosinolates, cyanogenic glycosides, oxylipins, and alkaloids [46]; there is a thriving interest in natural ingredients with multifunctional properties in food as well. Most edible plant parts include trace amounts of plant defense substances (phenolic acids), categorized as hydroxybenzoic and hydroxycinnamic acids [109]. Hydroxybenzoic acids are frequently found in larger phenolic compounds, such as hydrolyzable tannins. Hydroxycinnamic acids occur as esters of glycerol, tartaric, shikimic, and quinic acids, as well as glycosylated derivatives [110]. Plant extracts can inhibit harmful bacteria from adhering to the host cell membrane. As a result, it reduces bacterial attachment to host cell surface membranes, and thus sometimes it becomes a potential anti-adhesive agent [143].

Study [111] investigated the antifungal efficacy of different raisin extracts and by-products in traditional bread. Compared to a sample containing no preservatives, the bread produced with raisin paste and raisin water separate (7.5%) exhibited the best mold-reducing abilities. Leaf extracts of cherry laurel (*Prunus laurocerasus* L.) were recommended as potential bio-preservatives after demonstrating a very low MIC (mg/mL) against a variety of bread spoilage fungi [112]. Further, [113] showed that bread produced with a mix of sourdough and pea flour hydrolysate fermented by the antifungal strain *Lb. plantarum* 1A7 had the most extended shelf life. It is also effective against *P. roqueforti* DPPMAF1. Moreover, bread formulations containing free or liposome-encapsulated garlic extract (0.65 mL/100 g of dough) were found to be more microbiologically stable than controls, inhibiting molds such as *P. herquei*, *F. graminearum*, and *A. flavus* for five days [12].

### 6.3. Essential Oil as Bio-Preservative

Plant essential oils are receiving much attention in the food sector because of their potential as decontaminating agents, food flavoring agents, and natural food preservation agents [114]. They are also GRAS (Generally Recognized as Safe) [115]. EOs are formed from the largest category 1-phytoanticipins, intended antimicrobial elements found in plants and applied in the food, pharmaceutical, agronomic, and cosmetic industries [116]. The other categories include (cat. 2) inducible preformed compounds and (cat. 3) phytoalexins, which comprise activated restraining substances when the plant is attacked by a pathogen [117]. These substances can be found in the skins, shells, bark, and cereal bran of fruits, vegetables, and plants [110].

Numerous research has been conducted to figure out the efficacy of essential oils in enhancing the shelf life of bread (Table 4). Carvacrol and eugenol, two antifungal components found in essential oils, could be regarded as powerful antifungal agents. The inner mitochondrial membranes of fungal cells can be largely destroyed, while the cell wall is completely destroyed by them [114]. Thus, essential oils work as antifungal agents. The effects of thyme, clove, cinnamon oils, and orange, sage, and rosemary oils on rotting fungi in rye bread have been examined. Oils of thyme, clove, and cinnamon were known to suppress spoilage fungi, but oils of orange, sage, and rosemary had only a minor impact [118]. Among them, the potential of using marjoram and sage essential oils on bread is neglected owing to their low acceptability in terms of flavor and odor, despite their demonstrated mold inhibiting capabilities [119]. Study [120] used a disc diffusion experiment to study the antifungal effect of several EOs and reported that cinnamon and mustard EOs might cause a 100% inhibition in *P. roqueforti* multiplication with only 1 µL of EO introduced into the Petri dish system. In comparison, eugenol in cinnamon EO [121] and allyl isothiocyanate in mustard EO are the primary antifungal active components [120]. Furthermore, [122] investigated the antifungal activity of thyme essential oil in par-baked bread (0, 15, and 30 g sourdough/100 g dough) using the macro-dilution method with changed pH (4.8, 5.0, 5.5, and 6.0), a_w_ (0.95 and 0.97), and temperature (22 and 30 °C). Despite thyme oil′s strong in 1vitro potential, there was no noticeable shelf-life expansion for par-baked bread. In the case of rosemary oil, [123] found that applying 50.0 μL/mL rosemary essential oil inhibited both the *Penicillium* sp. and the *Aspergillus* sp. fungi tested. After 8 days of preservation at 25 °C, the amount of fungal generation in the dough containing pure oil and the dough containing microencapsulated oil decreased by at least 0.7 and 1.5 log cycles, respectively, in comparison to the control. A study by [124] evaluated essential oils of oregano (Origanum vulgar) and clove bud (*Syzygium aromaticum*), which were processed using low-speed mixing and ultrasonication to create coarse emulsions (1.3–1.9 μm) and nanoemulsions (180–250 nm). The results showed that both essential oils significantly reduced yeast and mold counts in sliced bread during 15 days. Apart from this, bread was treated with lemongrass EO to the amount of 125 to 4000 μL/L_air_ where *P. expansum* generation was inhibited for 21 days at 20 °C [125].

Although using essential oils in the bread industry has many benefits, one major downside is that the consumer does not always appreciate the flavor and aroma they impart. Article [126] documented color changes in food because of essential oils.

**Table 4:** Preservation of bread by plant essential oils.

Essential Oils	Targeted Molds	Results	Reference
Thyme	*Aspergillus niger* *P. paneum*	No noticeable shelf-life extension	[122]
Lemongrass	*P. expansum*	Mold growth was inhibited for 21 days	[125]
Rosemary	*Penicillium* sp. *Aspergillus* sp.	Fungal generation reduced by 0.7 and 1.5 log cycles after using pure rosemary oil	[123]
Clove bud and Oregano	*A. niger* *Penicillium* sp.	Reduced yeast and mold growth for 15 days	[124]
Marjoram and clary sage	*P. chrysogenum* *Rhizopus* spp.	Shelf life 8 days	[127]
Citrus peel	*General fungi*	Shelf life 4 days	[128]
Cinnamon and mustard	*P. roqueforti*	100% reduction of the targeted mold growth	[120]

### 6.4. Animal-Derived Products

Cheesemaking generates a waste stream with a high biochemical oxygen demand, whey, which is the liquid fragment produced after milk protein coagulation and is also a contaminant to the environment [129]. In recent times, there has been a gush of attraction in investigating and promoting natural antimicrobial compounds produced from food industry by-products that prevent the production of fungi in food [103]. The antimicrobial or antifungal compounds cause target cell membranes to permeabilize, resulting in holes, cell leakage, and cell death [148]. This two-pronged strategy addresses the health problems accompanied by chemical food additives by redressing them with natural preservatives, thereby encouraging better food items and contributing to the prevention and reduction of food waste [130,131]. Whey can be an intriguing technique for bread bio-preservation. A study conducted by [132] exhibited that whey’s application as a bio-preservation agent in bread improved shelf life by almost 2 to 15 days compared to bread that contained 0.3% calcium propionate and controlled untreated bread. Bread produced with goat whey hydrolysate (HGW) and treated with toxigenic fungi was included in a shelf life study by [133]. This study determined the effect of calcium propionate on fungal growth and mycotoxin formation in bread. It was proven that bread containing HGW inhibited fungal growth, with minimal inhibitory and fungicidal concentrations of 3.9–62.5 and 15.8–250 g HGW/L, respectively. In addition, HGW showed a 1-log reduction in fungal production, 85–100% mycotoxin generation, and a 2-day shelf life extension.

### 6.5. Nanoparticles

Efforts to provide effective bioactive packaging action and to prevent most biopreservatives from degradation under harsh conditions, including high temperatures and high humidity, may improve bakery products by implementing nanotechnology into the food business, specifically by incorporating nanomaterials [75].

Diseta et al. [134] carried out a recent experiment to assess the antifungal efficacy of nanocomplexes based on egg white protein nanoparticles (EWPn) and carvacrol (CAR), bioactive compounds (BC), trans-cinnamaldehyde (CIN), and thymol (THY), as well as their use as edible coatings on preservative-free bread. It was found that EWPn-CAR and EWPn-THY nanocomplex coatings had higher antifungal efficacy, allowing the bread to last an additional 7 days after application. Another study by [75] evaluated starch/carvacrol nanofibers where nanofibers containing 30 or 40% carvacrol showed restriction zones with limited generation and were successful in suppressing both fungi tested in the study. Besides the fact, only bread evaluated with starch/carvacrol nonwovens with 30% carvacrol had lower CFU values and no fungal development after 7 days (0 CFU). As well, incorporating nanomaterials into chitosan-based food wrapping techniques can help to inhibit spoilage and pathogenic microorganism generation, enhance food quality and safety, and lengthen the food shelf life. Based on the findings of study [135], it appears that chitosan-based films, coatings (or treatment) have been applied to prolong the shelf life of fresh produce, meat, bread, and dairy products. It could be a novel food packing system [136]. More recently, [137] studied the potential of expanding the shelf life of white bread by employing paper packages modified with Au/TiO_2_, Ag/N-TiO_2_, and Ag/TiO_2_-SiO_2_. They discovered that packaging with Ag/N-TiO_2_, and Ag/TiO_2_-SiO_2_ paper prolonged the shelf life of bread by 2 days, while utilizing Au/TiO_2_ paper had no effect.

However, though nanoparticle-based packaging materials are increasing its wide acceptance, there are still possibilities of migrating nanoparticles from packaging materials to foodstuff. Considering human health effects, it is also essential to consider short- and long-term toxicity studies. Nevertheless, it is not so easy to predict the NPs’ mode of action due to their vast range of physicochemical and biological behaviors [145]. Different countries are taking several robust regulatory approaches to cover all these issues. In the US, FDA (Food and Drug Admininstration) looks into the size of the NPs ranging from 1 nm to 100 nm, external dimension(s) (up to 1 µm), properties, etc. [146]. Even in Europe, stakeholders are demanding for greater transparency by either the labeling of products containing NPs or making use of nanotechnology [147].

### 6.6. Other Novel Technologies

Dispersion from the wrapping material to the food surface is a significant issue when it comes to the choosing and employment of plastic packaging substances for food packaging (2002/72/EC) [138]. The initial purpose of food packaging is to denounce the reactions that deal with the durability of the contents enclosed. Also, effective packaging selection and optimization are critical for food manufacturers.

Nowadays, various packaging materials with varying barrier qualities are available for food packaging, making the problem of selecting the best packaging material for a specific food product more challenging than ever. We will be discussing a few novel technologies of bread preservation below.

The existing packaging materials serve as a barrier to protect the bread from an adverse environment and any spoilage. Among several novel technologies for bread preservation, active packaging can either increase or observe the shelf life by actively interacting with it, which usually necessitates the application of chemical compounds [139]. Active packaging methods reduce bread spoilage by utilizing ethanol emitters, essential oil, and oxygen absorbers with other antimicrobial factors as a coating in the packaging or edible films, or by inserting them into the packaging, for example, as sachets [46,139]. They offer several benefits, along with the capacity to control the inner conditions of the package headspace, the partial or total distribution of other chemical preservatives, as well as the expansion of mold-free shelf life and the conservation of good sensory attributes for more extended periods, allowing for faster stock rotation cycle times and the extension of the distribution channel for bread item distributors [11]. Moreover, [140] studied the influence of active packaging with a cinnamon essential oil label mixed with MAP on the shelf life of gluten-free sliced bread in 2011. They discovered that active packaging extended the shelf life of packed food while retaining the gluten-free bread’s sensory qualities.

The rising consumer concern about food preservatives is prompting an expansion in demand for preservative-free goods. Modified Atmosphere Packaging is a substitute to chemical preservatives for governing mold decomposition in bread items, aside from their a_w_ and pH, which is described as the process of enclosing a food item in a high gas resistance film with the gaseous environment altered or regulated to reduce respiration rate, lower microbiological development, and hinder enzymatic decomposition to extend shelf life [141]. This mechanism involves injecting nitrogen (N_2_) and carbon dioxide (CO_2_) into the environment where the food packaging is placed. These gases are generally incorporated to appreciate the shelf life of bakery items by preventing fungal growth [138]. To determine which gases are the most successful in maintaining freshness, [142] compared the shelf life of bread prepared and conserved under varying concentrations of gases. They found out that the combination of 50% CO_2_ and 50% N_2_, with and without calcium propionate, was most dynamic against mold and yeast growth, increasing the shelf life to 117% and 158% at 22–25 °C and 15–20 °C, respectively.

## 7. Future Aspects and Bio-Preservatives Use in the Food Industry

The fungus problem in bread is alarming, and controlling it is a massive challenge for businesses. In another scenario, the value of functional foods has risen significantly in recent decades, and it is foreseen to continue to grow in importance with the niche market’s demanding consumers. So, to resolve this demand for functional and preservative-free safe food, the ongoing experiment has concentrated on bio-preservation techniques to increase the shelf life of bread. While bio-preservation has a long-term positive impact on food, it also has certain limitations. The primary barrier to using essential oils (EO) in foodstuff is that the individual compounds of EOs are insufficient to prevent microbial growth. The antimicrobial activity of EO also depends on the application scheme and the matrix into which it is put.

Furthermore, while spraying EOs to the surface, the active ingredients may get oxidized or volatilized. In addition, the antifungal possibilities and binding abilities of mycotoxins are strain-specific despite the vast abundance of LAB. This should be considered when choosing LAB as starter cultures in foodstuff. Also, the use of antifungal starter strains is confined yet, and additional experimentation is needed to enhance the duration of shelf life of gluten-free bread. In the case of active packaging, adequate labeling information with microbiological ecology must be provided. The price of food products with active packaging must be controlled while keeping in mind consumer acceptances. Finally, adapting and scaling up these technologies for industrial production would be the most effective approach, with the potential to be employed in combination with other technological hurdles, along with safety evaluation and risk analysis initiatives.

## 8. Conclusions

The pH, preservatives, and a_w_, along with the microbial ecology of the food material, the redox state, the permeability attributes of the packing film, the gaseous status surrounding the product, and the storage temperature, are all interconnected factors in food deterioration and food safety. While new and evolving treatment processes can prolong the shelf life of bakery items, they should not endanger the safety of the products. As a result, the on-going study of the impact of these novel treatment methods on the safety and shelf life of high moisture bakery goods is mandatory at the government, industry, and academic levels. This review addressed some of these novel methods, which may be helpful to manage both the food safety and food spoilage problems. Moreover, depending on the information accumulated here so far, these results may emerge to be a breakthrough for producing natural, high-quality bread with slight allergenic components with extended shelf life.

## Figures and Tables

**Table 1:** Keywords used for literature search.

Primary Keywords	Secondary Keywords ^a^
Bio-preservation of bread Bread spoilage Staling of bread Bread preservation by natural anti-microbes Mold spoilage of bread	Nanoparticles incorporated into bread Bakery product storage life Spoilage control of bread Lactic acid bacteria used in bread preservation Statistics on bakery waste

^a^ Literature search was conducted by using primary keywords in combination with secondary keywords.

**Table 2:** Significant microbes responsible for microbial bread spoilage.

Major Spoilage Concerns	Spoilage Agents	Influencing Factor/Species	Properties of The Microbe’s Colony	Issues	Reference
Physical	Staling	Starch retrogradation Moisture distribution Protein Baking process Storage condition		Crust softening Crumb firming	[51,55]
Chemical	Rancidity	Lipid Oxygen Short-chain fatty acid aldehyde		Off-flavors Off-odors Loss of vitamin (A,E) Protein degradation	[54]
Microbial	Bacteria	*Bacillus subtilis*	Irregular shape	Rotten fruit odor Sticky breadcrumbs Protein degradation Black discoloration	[7,60,65]
		*Bacillus licheniformis*	White/dull color		
		*Bacillus amyloliquefaciens*	Gram-positive		
		*Bacillus pumilus*	Ellipsoidal		
	Mold	*Rhizopus nigricans*	Grey, fluffy, fast spread	Mycotoxin Product loss Chalk mold	[66,67,68,69]
		*Penicillium expansum*	Blue/green, slow spread		
		*Aspergillus niger*	Black, fluffy, sporehead		
		*Cladosporium*	Dark green, flat, spreads slowly		
	Yeast	*S. cerevisiae*	Rapid growth, thermotolerance	Chalky bread White dot on bread Esteric off-odor	[46,70,71,72]
		*H. burtonii* *Scopsis fibuligera* *P. anomala*	Slow growth, low, white, spreading colonies		

**Table 3:** Preservation technique by microbial fermentation to improve bread shelf life.

Microbes	Product	Starter Culture Used, Compounds	Shelf Life/Fungal Inhibition	Reference
Lactic acid bacteria	Pan bread	*Lactobacillus plantarum*	7 days after baking, *A. niger* growth was lower	[98,99,100]
	Quinoa and rice bread	*Lactobacillus reuteri*, *Lactobacillus brevis.*	2 days extended shelf life	[93,101,102,103]
	Bread	*Lactobacillus plantarum*	>14 days extended shelf life	[104,105,106,107]
	Gluten-free breads	*Lactobacillus amylovorus*	4 days extended shelf life	[99]
	Bread	*P. acidilactici* KTU05-7, *P. pentosaceus* KTU05-8, and KTU05-10	8 days fungal growth inhibition	[100]
	Bread	*Lactobacillus hammesii*	6 days extended mold-free shelf life.	[108,109,110,111]
	Bread	*Lactobacillus plantarum* 1A7	Up to 28 days fungal inhibition	[104]
Yeast	Pan bread	*Penicillium anomala* SKM-T	Overall storage life is 6–8 days, when appearing with fewer fungi count	[105]
	Wheat sourdough	*W. anomalus* LCF1695	Up to 14 days shelf life	[104]

## Data Availability

Not applicable. The data used to generate the results of this study are included in the article and no additional source is required.
